# Unbalanced distribution of materials: the art of giving rise to hepatocytes from liver stem/progenitor cells

**DOI:** 10.1111/jcmm.12183

**Published:** 2013-11-28

**Authors:** Wei-Hui Liu, Li-Na Ren, Tao Chen, Nan You, Li-Ye Liu, Tao Wang, Hong-Tao Yan, Hao Luo, Li-Jun Tang

**Affiliations:** a General Surgery Center of PLA, Chengdu Military General HospitalChengdu, Sichuan Province, China; b Department of General Surgery Xinqiao Hospital, Third Military Medical UniversityChongqing, China

**Keywords:** liver stem/progenitor cells, hepatic differentiation, unbalanced distribution theory, hepatocytes, liver regeneration

## Abstract

Liver stem/progenitor cells (LSPCs) are able to duplicate themselves and differentiate into each type of cells in the liver, including mature hepatocytes and cholangiocytes. Understanding how to accurately control the hepatic differentiation of LSPCs is a challenge in many fields from preclinical to clinical treatments. This review summarizes the recent advances made to control the hepatic differentiation of LSPCs over the last few decades. The hepatic differentiation of LSPCs is a gradual process consisting of three main steps: initiation, progression and accomplishment. The unbalanced distribution of the affecting materials in each step results in the hepatic maturation of LSPCs. As the innovative and creative works for generating hepatocytes with full functions from LSPCs are gradually accumulated, LSPC therapies will soon be a new choice for treating liver diseases.

- Introduction
- The identification of cells generated from LSPCs
  –Morphological characteristics
  –Phenotypic markers
  –Functional evaluation
  –In vivo transplantation
- Strategies for inducing the hepatic differentiation of LSPCs
  –Modification of the physical parameters in LSPC cultures
  –The induction of LSPCs by adding feeder cells
  –Addition of soluble medium factors
  –The induced differentiation of LSPCs on bio-scaffold
  –Determination of cell fate via epigenetic modification
- Detailed mechanisms of hepatic differentiation from LSPCs
  –The initiation of hepatic differentiation
  –The progression of hepatic differentiation
  –The accomplishment of hepatic differentiation
- Conclusions and perspectives

## Introduction

Liver transplantation is currently the most effective treatment for liver failure, but its use is limited by the scarcity of organs for transplantation, the high cost, and lifelong immunosuppression necessary after transplantation. Cell-based therapy is a promising strategy to solve these problems. Studies on liver stem/progenitor cells (LSPCs) have shown especially promising results for overcoming the above-mentioned limitations 1. Liver stem/progenitor cell therapy has potential advantages in several aspects over liver transplantation 2–3: (*i*) LSPCs have great potential for generating numerous functional cells in the liver such as mature hepatocytes and cholangiocytes; (*ii*) LSPCs are present in the liver regardless of donor age#x2014;they are present in the ductal plates in foetal and neonatal livers and in the Canals of Hering in paediatric and adult livers 4; (*iii*) LSPCs with liver fate specification have been isolated and identified, including foetal liver stem/progenitor cells (FLSPCs, also called hepatoblasts) and adult liver stem/progenitor cells (ALSPCs, including oval cells #x005B;OCs#x005D; and small hepatocytes #x005B;SHs#x005D;); (*iv*) LSPCs can be genetically manipulated, implanted without major surgery and cryopreserved for future use; (*v*) the requirements for immunosuppression are not strict in most LSPC transplantation cases.

In addition to the promising therapeutic effects for liver failure, LSPCs also play a critical role in liver regeneration after severe liver damage 5. Liver regeneration can be defined as a three-stage process of cell replacement. The first stage is characterized by an ability of mature hepatocytes to undergo rapid proliferation to regenerate the liver in response to certain types of injuries. Although normally proliferatively quiescent, hepatocytes can undergo a rapid regenerative response to restore liver mass that is induced by hepatocyte loss, which is commonly caused by partial hepatectomy (PHx). This restoration of moderate cell loss and #x2018;wear and tear#x2019; of renewal is largely achieved by hepatocyte duplication 6. However, when the liver suffers from severe and/or chronic damages and hepatocyte proliferation is delayed or suppressed, LSPCs are activated 7–8. This second stage of liver regeneration is characterized by the participation of an intrahepatic stem cells. The best proof comes from various human analyses and animal models of extensive hepatic damage, in which the proliferating LSPCs from the Canals of Hering differentiate towards the hepatocytic and cholangiocytic lineages according to the severity of the disease and the type of mature epithelial cell that is damaged 9. It is under these conditions that the facultative LSPCs show up and take part in the liver regeneration process 10,11. The third stage of liver regeneration is believed to involve the participation of an extrahepatic cell source that consists of cells coming from the circulation. The cells are most likely of bone marrow origin, although derivation from other sources has not been excluded.

Despite the fact that LSPCs are promising for cell therapy and are essential for liver regeneration, the fundamental problem in clinical applications lies in generating mature functional cells from LSPCs 12. Among the many types of cells consisting of the liver, hepatocytes are the most important epithelial cell lineage 13. Thus, the first core issue for the application of LPSCs is how to efficiently promote their differentiation into hepatocytes. Unfortunately, the degree of maturation of the cells induced from LSPCs may not be equal to that of the healthy resident hepatocytes, and the details of the hepatic differentiation process are not well understood. *In vitro* culture systems as well as *in vivo* studies have elucidated detailed molecular mechanisms, including intercellular signalling networks and intracellular transcriptional regulatory webs, that co-ordinately regulate the hepatic differentiation of LSPCs. Understanding the cellular and molecular bases of hepatic differentiation from LSPCs will be invaluable in producing fully functional hepatocytes that can be applied for cell therapy and pharmaceutical screening in the future 14.

In this survey, we also provide an up-to-date overview of the wide variety of experimental conditions that have been applied thus far to trigger the differentiation of cultured LSPCs into hepatocytes. In principle, most approaches are based on reconstructing the *in vivo* microenvironment *via* (*i*) reconstitution of the cell matrix, (*ii*) cell–cell interactions (feeder cells), (*iii*) addition of soluble medium factors, (*iv*) chromatin modulation, (*v*) overexpression of liver-enriched transcription factors (LETFs), and (*vi*) treatment with biomaterials. No matter how the differentiation of LSPCs is triggered, we hypothesize that the unbalanced distribution of materials such as inductive molecules, facilitative signalling pathways and the accompanying LETFs, is crucial in deciding the cell fate of LSPCs (Fig.#x00A0;1). In this review, we revisit landmark studies, summarize the current nomenclature, and discuss recent data that elucidate potential methods and mechanisms of hepatic differentiation from LSPCs. The characterization of the molecular and cellular events accompanying the hepatic differentiation of LSPCs is essential for understanding the basic biology of LSPCs and for facilitating the clinical application of these stem cells.

**Figure 1:**
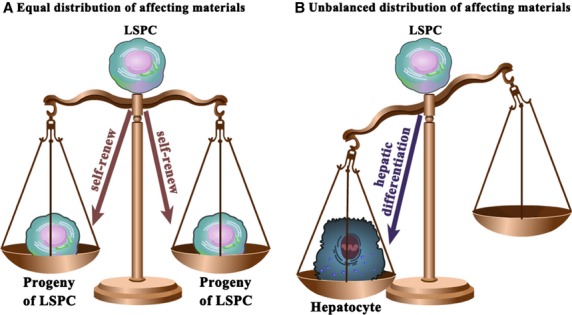
The balance theory for the cell fate decisions of liver stem/progenitor cells (LSPCs). (A) When the LSPC daughter cells inherit equal material distribution, they will potentially present the same characteristics as the mother cells. This is the so-called symmetrical division of LSPCs that allows for the self-renewal of LSPCs. (B) However, if some stimuli result in an unbalanced distribution of materials from the mother LSPCs to the daughter cells, the LSPCs will be prone to undergo asymmetric division. As a result, the LSPCs will differentiate into mature cells such as hepatocytes. Whether the LSPCs can give rise to functional hepatocytes depends on the type of material basis the progeny acquire from the mother LSPCs.

## The identification of cells generated from LSPCs

This section discusses how to identify undifferentiated LSPCs and induced hepatocytes. The following criteria are discussed (Fig.#x00A0;2): (*i*) typical morphological characteristics; (*ii*) expression of lineage-specific markers; (*iii*) essential functional achievements; (*iv*) *in vivo* transplantation for cell therapy. We must identify the cell fate of LSPCs according to the features of the primitive resident cells comprising the liver.

**Figure 2:**
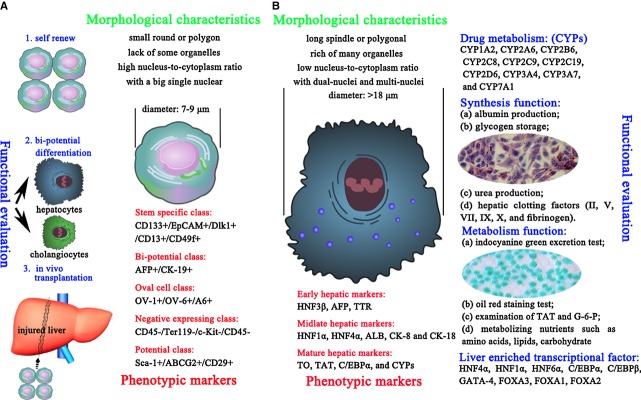
The identification criteria for the cell fate of liver stem/progenitor cells (LSPCs). (A) The undifferentiated LSPCs should contain three main properties: stem-like morphological characteristics, expression of specific markers and functional capacities of self-renewal, multipotent differentiation and rescue of injured liver tissues. (B) The typical differentiated hepatocytes should also be judged using the aforementioned three aspects. In addition to the typical cuboid morphology, attention must be paid to the essential functions of mature hepatocytes. These functions involve protein synthesis, protein storage, the transformation of carbohydrates, the synthesis of cholesterol, bile salts and phospholipids, and the detoxification, modification and excretion of exogenous and endogenous substances.

### Morphological characteristics

#### The morphology of LSPCs

Liver stem/progenitor cells are characterized by their uniform morphology, high nucleus-to-cytoplasm ratio, small size (7–9#x00A0;#x03BC;m in diameter) and tightly packed colony formation 3. Because LSPCs have specific morphological characteristics, Liu *et#x00A0;al*. can establish a three-step method to isolate such LSPCs from foetal liver 15.

#### The morphology of induced hepatocytes

When LSPCs differentiate into hepatocytes, the cells grow in size to >18#x00A0;#x03BC;m and display a cordlike colony morphology. These induced cells can form hepatic plate-like structures consisting of long spindle or polygonal epithelia-like cells with dual-nuclei or multi-nuclei when viewed under a light microscope. In ultrastructural studies, these cells acquired typical hepatocyte features such as large numbers of mitochondria, rough endoplasmic reticulum (ER) and Golgi complexes.

### Phenotypic markers

The differentiation of LSPCs into mature cells is a steady process, and stem cells, progenitor cells and mature cells differ in their molecular phenotypes 16–17.

#### The typical markers of undifferentiated LSPCs

Although great progress has been made in the isolation and identification of LSPCs, there are still no widely accepted markers that are specific for LSPCs. The reason for this is twofold: (*i*) LSPCs themselves show a wide range of phenotypic heterogeneity; (*ii*) many of the currently used markers remain controversial among different research groups. Thus, in this review, we present the markers that are the most effective for analysis.

In general, four markers are the most widely used for identifying FLSPCs. The widely used markers include cluster of differentiation (CD) 133 (also known as Prominin-1) 15–18, epithelial cell adhesion molecule (EpCAM) 19–20, Delta-like 1 homolog (Dlk1; also known as Pref-1 or foetal antigen 1) 21–24, CD13 (aminopeptidase N) 25. To avoid false positives, some groups have tried using combinations of the above markers to isolate FLSPCs such as CD13 and CD133 26–27, EpCAM and Dlk1 28, EpCAM and CD133 29–30. In addition to the previously mentioned markers, some lesser-known markers are used for FLSPC isolation and identification, such as Stem cell antigen-1 (Sca-1) 31, E-cadherin 32, intercellular adhesion molecule-1 (ICAM-1) 33. Several groups believe that FLSPCs should express multiple markers, including alpha6- and beta1-integrin subunits (CD49f and CD29, respectively), and they have proven that the CD45-/TER119-/c-Kit-/CD29#x002B;/CD49f#x002B; and the CD45-/TER119-/c-Kit-/c-Met#x002B;/CD49f#x002B; fractions of foetal liver cells are FLSPCs 34–37.

Compared to FLSPCs, older and more numerous research studies have identified markers of ALPSCs. Until now, several monoclonal antibodies have long been used as the #x2018;golden standards#x2019; to identify OCs (representative ALSPCs) including oval cell 1 (OV-1) and OV-6 in rats 11 and A6 in mice 38, which has resulted in the identification of other widely used markers. Similar to FLSPCs, the previously mentioned four markers of FLSPCs (CD133, EpCAM, Dlk1, CD13) are also applicable in identifying ALSPCs 4–41. It is also necessary to combine the above markers together to accurately isolate ALSPCs. Using flow cytometric analyses for over 90 antigens, Kakinuma *et#x00A0;al*. demonstrated the pure enrichment of ALSPCs using combinations of several positive markers, including CD13, Dlk1 and CD133 25. Other combinations tested include CD13, CD49f and CD133 26–27.

Adult liver stem/progenitor cells show a wide range of phenotypic heterogeneity, and more and more new molecules have been reported to be ALSPC markers, including c-kit 7–17, Thy1 42,43, Sca-1 45, the transcription factor forkhead box L1 (Foxl1) 46, and the oncofoetal protein glypican-3 47. Notably, the triphosphate-binding cassette transporter, ATP-binding cassette sub-family G member 2 (ABCG2)/breast cancer resistance protein 1 (BCRP1), a marker for the side population (SP), is also a putative marker for ALSPC isolation 48. Based on this hypothesis, Liu *et#x00A0;al*. have successfully isolated ALSPCs by SP from injured rat liver 49. Adult liver stem/progenitor cells are thought to be capable of differentiating into two liver epithelial lineages, the hepatocyte and cholangiocyte lineages. Therefore, ALSPCs should express both early hepatocyte and early cholangiocyte markers. Thus, FACS using stem/progenitor cell markers such as CD133 and EpCAM in combination with lineage markers such as a-foetoprotein (AFP) and cytokeratin 19 (CK19) to enrich ALSPCs would be more effective 50,51. However, caution should be taken for different species. For example, the early hepatic marker AFP is known to be expressed in ALSPCs in rats but not in mice 43. In addition, CK19 seems to be absent in human ALSPCs 53.

In summary, when judging whether LSPCs differentiate or not, keep the following in mind: (*i*) select the most widely accepted markers; (*ii*) combine more than two markers; (*iii*) pair stem cell markers with early liver lineage markers; and (*iv*) consider the species of LSPC origination.

#### The specific markers of differentiated hepatocytes

Hepatogenesis *in vivo* involves the serial expression of early markers (hepatocyte nuclear factor #x005B;HNF#x005D;3#x03B2;, AFP and transthyretin #x005B;TTR#x005D;), mid/late markers (HNF1#x03B1;, HNF4#x03B1;, albumin #x005B;ALB#x005D; and CK18) and late markers (tryptophan 2,3-dioxygenase #x005B;TO#x005D;, tyrosine amino transferase #x005B;TAT#x005D;, Ccaat-enhancer-binding protein (C/EBP) #x03B1; and cytochrome P450 #x005B;CYPs#x005D;) 54–55. Accordingly, during hepatic differentiation *in vitro*, cells sequentially converted from expression of early markers (HNF3#x03B2;, AFP, and TTR) to mid/late markers (HNF1#x03B1;, HNF4#x03B1;, ALB, and CK18) to late markers (TO, TAT, C/EBP#x03B1;, and CYPs) 54–56. In other words, during hepatic differentiation, the daughter cells of LSPCs will gradually lose their expression of stem cell markers and slowly gain expression of hepatocyte markers such as AFP, ALB and CK18. The most studied endodermal markers include the LETFs (HNF1#x03B1;,#x03B2;, HNF3#x03B2;, HNF4#x03B1; and C/EBP#x03B1;,#x03B2;), plasma proteins (AFP, ALB, TTR) and cytoskeletal proteins (CK18, CK8). A minority of studies have also examined the expression of the CYPs (CYP2C8, CYP2C19, CYP3A4, CYP3A5, CYP3A7, CYP7A1) and other #x2018;late#x2019; enzymes such as TO, TAT and glucose-6-phosphatase (G-6-P) 57.

### Functional evaluation

#### The usefulness of LSPCs

The main characteristic of stem cells is self-renewal and multipotent differentiation (Fig.#x00A0;2A). Therefore, LSPCs can be identified in terms of clonal growth, maintenance of stemness and the acquisition of differentiation under specific inductive conditions. In other words, LSPCs should rapidly duplicate themselves during *in vitro* culture and after induction should be able to generate ALB-positive hepatocytes and CK-7-positive cholangiocytes. In addition, as mentioned above, LSPCs should be able to functionally reconstitute the liver parenchyma efficiently after injury.

#### The certification of hepatic function

We first need to establish a list of mature hepatic functions that can be easily measured. In other words, we need fast and easy tests that provide relevant and robust information on the hepatic capabilities of the LSPC-derived hepatocytes. From a functional point of view, any candidate hepatocyte-like cell type should represent a minimal set of hepatic functions of a true hepatocyte 58. Here, we present a battery of relevant studies for the analysis of the functional activities of LSPC-derived hepatocytes: (*i*) expression analysis of genes found in mature hepatocytes, such as LETFs and CYPs; (*ii*) metabolism of xenobiotics and endogenous substances (the indocyanine green #x005B;ICG#x005D; excretion test, the oil red staining test, and the examination of TAT and G-6-P); (*iii*) synthesis and secretion of ALB, clotting factors, transporter proteins, bile, lipids and lipoproteins; and (*iv*) storage of glucose (glycogen), folate, vitamin B12, copper, iron and the fat-soluble vitamins A, D, E and K. Detailed information on the essential aspects can be found in Figure#x00A0;2B.

### *In vivo* transplantation

As we have mentioned in our previous articles, the *in vivo* identification of differentiated cells is essential, and in some sense it is the #x2018;golden standard#x2019; of certifying the cell fates of differentiated LSPCs 59. The transplanted LSPCs should be able to functionally reconstitute the liver parenchyma (including both hepatocytes and cholangiocytes) efficiently after injury (Fig.#x00A0;2A). Accordingly, a convincing *in vivo* experiment to confirm the identity of LSPC-derived hepatocytes is to restore damaged hepatocytes and recover liver function in animal models. To monitor the implanted cells and their contributions, the transplanted cells should be labelled with either fluorescent materials 49 or with indium-111 ((111)In)-oxine and technetium-99 m ((99 m)Tc)-Ultratag or (99 m)Tc-Ceretec 60.

## Strategies for inducing the hepatic differentiation of LSPCs

Hepatocytes obtained from LSPCs and other stem/progenitor cells have not yet matured to the stage at which they can efficiently repopulate the liver of an adult. In other words, to use LSPCs in regenerative medicine, an effective procedure to accomplish hepatic maturation from LSPCs must be developed. The methods for controlling the cell fate of LSPCs can be determined from the *in vivo* microenvironment of LSPCs 61–62, which is composed of mesenchymal cells, as well as other cells, and extracellular matrices (ECM) that regulate the appropriate cell fate decisions of LSPCs. That is to say, most of our *in vitro* strategies for inducing hepatic differentiation of LSPCs come from monitoring the *in vivo* microenvironment. In other words, when it is needed to promote transplanted LSPCs or resident LSPCs to differentiate into hepatocytes, we only have to rebuild the required microenvironment for hepatic differentiation, including addition of some necessary materials. The co-ordinated signalling between stem cells, non-stem niche cells and the scaffold and the integration of stem cell-autonomous characteristics, including a dynamic interplay between transcription, epigenetic control and post-transcriptional regulators, represent an interactive system organized to facilitate cell fate decisions in a spatiotemporal manner 16. Taken together, there are two important rules for inducing the differentiation of LSPCs. On one hand, the differentiation of LSPCs is a gradually processing event, which requires caution to guarantee that cells pass through each stage smoothly. For example, it has been demonstrated that OCs first change into SHs before differentiating into mature hepatocytes 63. On the other hand, in our opinion, the unbalanced distribution of stimulating materials/factors, signalling pathways and expressed genes/proteins decide the cell fate of LSPCs. In light of these factors, the strategies for inducing LSPCs can be divided into the following categories (Fig.#x00A0;3).

**Figure 3:**
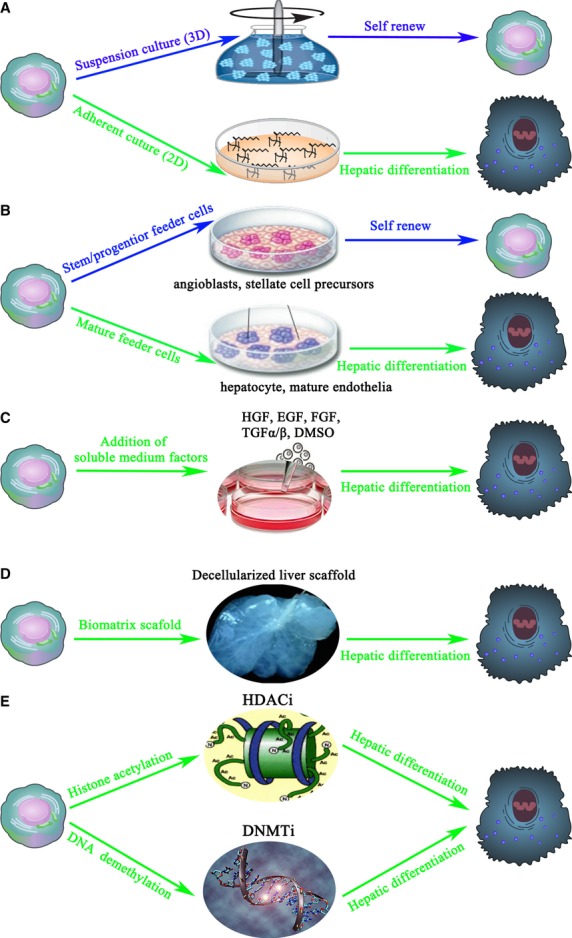
The inductive strategies of hepatic differentiation from liver stem/progenitor cells (LSPCs). The five main classes of inductive methods include (A) changing the physical parameters of LSPC culture, (B) co-culturing with mature feeder cells such as hepatocytes and endothelial cells, (C) adding inductive soluble medium factors consisting of growth factors and chemicals, (D) placing LSPCs on a biomatrix scaffold, (E) modifying the chromatin of LSPCs. HDACi, histone deacetylase inhibitor; DNMTi, DNA methyltransferase inhibitor.

### Modification of the physical parameters in LSPC cultures

In recent years, simulated microgravity, a physical force, has been shown to differentially regulate the proliferation and differentiation of stem cells. Microgravity, which is simulated using indigenously fabricated three-dimensional (3D) clinostats, can induce the differentiation of stem cells within 2–3#x00A0;days. Furthermore, microgravity interplays with signalling pathways (such as Wnt/Notch) in stem cells, thus better inducing the differentiation of LSPCs into hepatocytes 64. Based on our experiences, the manner of culture will also affect the cell fate of LSPCs (Fig.#x00A0;3A). When FLSPCs were grown in 3D suspension culture, they were likely to self-renew; in contrast, FLSPCs that were maintained in 2D adherent culture were prone to differentiation 65. These data are consistent with others#x2019; report 66. In summary, the space and gravity factors are essential for deciding cell fates of LSPCs.

### The induction of LSPCs by adding feeder cells

*In vivo*, it is believed that the surrounding cells can play important roles in controlling the cell fate of LSPCs. For example, within the niche, macrophages use paracrine signalling to control cell fate of LSPCs *via* TWEAK (tumour-necrosis-factor-like weak inducer of apoptosis) and the Wnt signalling pathway. After hepatocellular injury, macrophages ingest hepatocyte debris and release Wnt, which promotes LSPC differentiation into hepatocytes 67. *In vitro*, LSPCs can be induced to differentiate by co-culture with feeder cells such as fibroblasts, hepatocytes and mesenchymal cells 68–72. The subpopulations of liver-derived mesenchymal cells, purified by immunoselection technologies, include 73 (*i*) angioblasts, (*ii*) myofibroblasts, (*iii*) hepatic stellate cell precursors, (*iv*) mature stellate cells (pericytes) and (*v*) mature endothelial cells. In one word, if the feeder cells are stem-like cells, they will probably induce LSPC proliferation; however, if they are mature cells, they are more likely to induce LSPC differentiation (Fig.#x00A0;3B). Feeders of angioblasts yield self-replicating LSPCs, stellate cell precursors induce lineage restriction in the LSPCs, mature endothelial cells induce differentiation into hepatocytes, and mature stellate cells and/or myofibroblasts induce differentiation into cholangiocytes. However, as adding feeder cells is complicated and can have unknown effects, the gradients secreted by feeder cells can be analysed so that the differentiation of LSPCs can be completed by adding essential components.

### Addition of soluble medium factors

In liver development, a number of soluble medium factors (growth factors #x005B;GFs#x005D;, cytokines, corticosteroids, hormones) and components of the ECM lead to the differentiation of LSPCs 74. Thus, it is possible to examine the direct effects of GFs and ECMs on LSPCs (Fig.#x00A0;3C). Hepatocyte growth factor (HGF), fibroblast growth factor (FGF), epidermal growth factor (EGF), stem cell factor (SCF) and transforming growth factor #x03B1;/#x03B2; (TGF#x03B1;/#x03B2;) might simultaneously play central roles in the activation, proliferation, maintenance and differentiation of LSPCs 75–79. The differentiation of LSPCs into either the biliary or hepatic lineages greatly depends on the types of GFs used. A study by our group revealed that HGF can only induce the early transition of ALB-negative LSPCs to ALB-positive hepatocyte-like cells. Furthermore, a study by our group revealed that the combination of HGF with dimethyl sulphoxide (DMSO) accelerated the hepatic differentiation of LSPCs 65. Based on these theories, many groups combine several types of GFs to efficiently induce LSPC differentiation. In the presence of combined GFs (50#x00A0;ng/ml HGF, 20#x00A0;ng/ml EGF, 10#x00A0;ng/ml FGF), FLSPCs showed typical characteristics of hepatocyte-like cells 80.

### The induced differentiation of LSPCs on bio-scaffold

In addition, biomaterials can potentially influence stem/progenitor cell proliferation and differentiation in both a positive and a negative way (Fig.#x00A0;3D). A strategy for the rapid and efficient differentiation of LSPCs into hepatocytes uses biomatrix scaffolds, which are tissue-specific extracts enriched in ECMs and the associated GFs and cytokines, in combination with a serum-free, hormonally defined medium (HDM) 81. The scaffolds maintain native histology, patent vasculatures, <1#x0025; of the tissue#x2019;s proteins, >95#x0025; of the tissue#x2019;s collagens and most of the collagen-associated matrix components, and physiological levels of matrix-bound GFs and cytokines. Liver stem/progenitor cells supported by these scaffolds differentiated to mature, functional hepatocytes in ∼1#x00A0;week and remained viable and stable with mature cell phenotypes for more than 8#x00A0;weeks. Another biomaterial strategy uses a new bioactive membrane made of PEEK-WC-PU, whose surface is grafted with nitrogen functionalities by means of NH(3) glow discharges; these NH(3) plasma-grafted PEEK-WC-PU membranes allowed for the hepatic differentiation of LSPCs 82.

### Determination of cell fate *via* epigenetic modification

Epigenetic events are also thought to play a predominant role in the acquisition and maintenance of the differentiated phenotypes of LSPCs *in vitro*. In fact, the progression from stem cells to their differentiated progeny is characterized by alterations in the epigenetic landscape of the gene regulatory and coding regions 83. Recent studies suggest that stem cells are maintained by the integrative regulation of gene expression patterns related to self-renewal and that differentiation is induced by epigenetic mechanisms such as histone (de)acetylation and DNA (de)methylation 84,85. In other words, epigenetic events, including covalent histone modifications and DNA methylation, are broadly acknowledged to play a fundamental role in the cell fate decisions of stem cells. More specifically, locus-specific modifications of histones and DNA progressively silence the transcription of pluripotent genes (euchromatic heterochromatic state) while activating the typical differentiated, lineage-specific genes (heterochromatic euchromatic state) 87. Two opposing enzymes, histone acetyl transferases (HATs) (recently also referred to as lysine (K)-acetyltransferases or KATs) and histone deacetylases (HDACs), determine the acetylation status of the lysine residues on the N-terminal histone tails extending out of the nucleosomes 88. The methylation patterns are established by DNA methyltransferases (DNMTs), which catalyse the addition of a methyl group derived from the methyl donor S-adenosyl methionine 89. In summary, gene transcription, which governs the maintenance of a cell#x2019;s differentiation status in stem cells, can be modified by targeting the expression of DNMTs and/or HDACs and increasing the chromatin accessibility of transcription factors to their target DNA. The most commonly used histone deacetylase inhibitors (HDACis) in LSPC cultures are DMSO and sodium butyrate, which can efficiently induce hepatic differentiation 90–91. Taken together, both HDACis and DNA methyltransferases inhibitors (DNMTis) are potent modulators of liver-specific functions and cellular contacts, and as such, could significantly contribute to the acquisition and maintenance of hepatocyte-specific phenotypes in culture (Fig.#x00A0;3E). The addition of HDACis and/or DNMTis to LSPCs that have been preferentially co-conditioned with hepatogenic GFs and cytokines is a potential strategy for driving differentiation programs, particularly for directing hepatic differentiation. However, genotoxic factors may have important consequences if the HDACi/DNMTi-treated hepatocytes are then used in cell therapy.

The transcriptional activation of LETFs critically decides the hepatic differentiation of LSPCs. In particular, the sequential expression of forkhead box protein A2 (Foxa2, HNF-3#x03B2;), HNF4#x03B1; and C/EBP#x03B1; induces a mature hepatocyte phenotype in an expandable LSPC cell line 92. Recently, microRNAs (miRNAs) have gained significant attention as regulators of a variety of biological processes, including maintaining stemness and guiding the differentiation of stem/progenitor cells 93. As such, miRNAs represent cell-autonomous genetic elements that can establish different differentiation pathways in LSPCs. MicroRNA-122 (miR-122) is specifically and abundantly expressed in adult liver, and it may also function to enhance the differentiation process of LSPCs. Overexpression of miR-122 in FLSPCs resulted in significantly up-regulated expression of the hepatocyte-specific genes that facilitate hepatic differentiation 94.

## Detailed mechanisms of hepatic differentiation from LSPCs

Although LSPCs are thought to differentiate into hepatocytes, the details of the differentiation process are not well understood. In the above sections, we described the conditions needed for LSPCs to differentiate into fully mature hepatocytes. In the present section, we will clarify the related mechanisms for inducing the hepatic differentiation of LSPCs. In other words, both positive factors and negative factors collectively regulate the process of hepatic differentiation from LSPCs. However, at present, most of the researches are concentrating on the positive factors, and there is lack of knowledge of negative factors responsible for hepatic differentiation. In this review, we mainly pay attention to the positive factors. Generally speaking, different positive factors are unequally assigned to progeny cells, resulting in distinct directions of LSPCs differentiation. The differentiation of LSPCs is a gradual process that can be divided into three main stages: initiation, commitment and maturation (Fig.#x00A0;4) 13,50. In the initiation stage, different stimulators may induce differentiation in different directions. In the commitment stage, distinct activated signalling pathways could bring about distinct cell type commitments, and in the maturation stage, the unbalanced expression of transcriptional factors can create different functional cells. In short, it is the unbalanced distribution of both extracellular and intracellular signalling pathways that determines the direction of hepatic differentiation of LSPCs. The present proven molecular mechanisms of hepatic differentiation come from both *in vitro* and *in vivo* researches. We could use these mechanisms to correct the abnormal liver development and rescue the insufficient liver regeneration. Meanwhile, the knowledge of molecular mechanisms of hepatic differentiation could also guide LSPCs transplantation-based treatment of severe hepatocytes loss.

**Figure 4:**
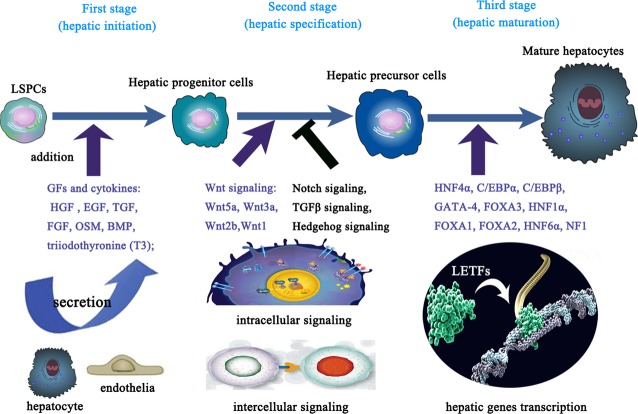
The molecular mechanisms in each step of hepatic differentiation of liver stem/progenitor cells (LSPCs). The hepatic differentiation of LSPCs can be divided into three main stages: initiation, specification and maturation. Several important growth factors and chemicals are thought to be responsible for initiating the first stage of hepatic differentiation. As hepatic differentiation continues, several key signalling pathways play crucial roles in guiding cells smoothly through the second stage. After the deciding liver-enriched transcription factors have been activated, the third stage of hepatic differentiation can be accomplished.

### The initiation of hepatic differentiation

The GFs are the most widely used materials to trigger the onset of hepatic differentiation of LSPCs (Fig.#x00A0;4). The identified GFs include HGF 95–96, TGF 97, FGF 98, Oncostatin M (OSM) 99, BMP 100 and triiodothyronine (T3) 101. In many cases, OSM, one of the interleukin 6-family cytokines, shows great potential for inducing the differentiation of LSPCs into functional hepatocytes 37–102. In addition to OSM, HGF has also been implicated as an important factor in stimulating hepatic differentiation 74. Because the maturation of LSPCs into the hepatocyte lineage is regulated by numerous factors, combining the above GFs can synergistically induce the hepatic differentiation of LSPCs. For example, the combination of HGF and OSM can more efficiently induce the hepatic differentiation of LSPCs 103–104. Recently, some new stimulators have been identified as initiating the hepatic differentiation of LSPCs, such as the polycomb group protein Ezh2 105 and S-Adenosylmethionine (abbreviated as SAM, SAMe or AdoMet) 106. Importantly, Ezh2-knockdown promoted the differentiation and terminal maturation of hepatocytes, followed by the up-regulation of several LETFs of hepatocyte differentiation 105. SAMe is known to be a key hepatic regulator. Recently, both *in vitro* and *in vivo* studies have demonstrated the regulatory role of SAMe in HGF-mediated hepatocyte proliferation *via* a mechanism that implicates the activation of the non-canonical LKB1/AMPK/eNOS cascade 106.

### The progression of hepatic differentiation

To date, the molecular signals regulating the differentiation of LSPCs are not fully understood. The Wnt family is essential for hepatic embryogenesis and is implicated in hepatic carcinogenesis 107–108. The canonical Wnt (Wnt/#x03B2;-catenin) signalling pathway is known to regulate the maturation, expansion and survival of FLSPCs, while the function of the non-canonical Wnt signalling pathway in LSPCs is currently unknown 109. Nevertheless, it is generally accepted that the down-regulation of the Wnt signalling pathway would result in the hepatic differentiation of LSPCs 107 through the downstream factor LEF1, which interacts with LETF and HNF4#x03B1; 110. Many members of the Wnt family play important roles in controlling the hepatic differentiation of LSPCs. In addition, Wnt1 has been found to direct the hepatic differentiation of OCs in the rat 2-acetylaminofluorene (2AAF) and 2/3 PHx liver regeneration models. In the absence of Wnt1 signalling, OCs failed to differentiate into hepatocytes and instead underwent atypical ductular hyperplasia, exhibiting epithelial metaplasia and mucin production 111. Similarly, Wnt5a-supplementation not only retards the formation of bile duct-like structures but also promotes the hepatic maturation of LSPCs *in vitro*; however, the loss of Wnt5a abnormally promotes the formation of bile duct-like structures in FLSPCs *in vivo* 112. In addition, Wnt2b and Wnt3a also contribute to the regulation of hepatic cell specification 113–114. Although there is much research on the Wnt regulation of LSPC differentiation, the exact roles of each Wnt member and the related underlying molecular mechanisms are far from complete. In spite of this, it is still believed that the Wnt signalling pathway is one of the most important signalling pathways for promoting the hepatic differentiation of LSPCs (Fig.#x00A0;4).

In addition to the Wnt signalling pathway, some other traditional signalling pathways also play important roles in controlling the hepatic differentiation of LSPCs. As Notch signalling is very important for regulating the cell fate of many types of stem cells, a study by our group tried to determine whether it is also essential for LSPC differentiation. As a result, the results confirmed that the inhibition of Notch signalling combining with HGF could efficiently induce hepatic differentiation of FLSPCs 115. Another study found that the Notch signalling member Notch3 may not only be a regulator of the hepatic differentiation of FLSPCs but also be a potential marker of FLSPCs 104. In addition, during hepatic differentiation, many downstream targets of TGF#x03B2; signalling, such as Smads (mothers against decapentaplegic homologue), are down-regulated 107. Hedgehog (Hh) signalling plays crucial roles in the development and homeostasis of various organs. The hepatic differentiation of FLSPCs *in vitro* could be significantly inhibited by the forced activation of Hh signalling 116.

### The accomplishment of hepatic differentiation

The core issue of hepatic differentiation is in the transcriptional changes induced by external stimuli and signals. In addition to the extracellular signals, hepatocyte differentiation and maturation are regulated by cell-intrinsic machineries involving various transcription factors (Fig.#x00A0;4). A set of transcription factors are known to be abundantly and characteristically expressed in hepatocytes and thus are collectively termed as #x2018;LETFs#x2019;; it has become evident that they function together to form a dynamic transcriptional network of autoregulatory and cross-regulatory loops 13–55. The intracellular transcription factors that regulate the expression of key mature liver proteins include HNF4#x03B1; 117–118, C/EBP#x03B1; 119, GATA binding protein 4 (GATA-4), forked box A 3 (Foxa3), HNF1#x03B1; 120, FOXA1, FOXA2 121, HNF6#x03B1; 122 and nuclear factor 1 (NF1) 123. Why are these LETFs so important for the hepatic differentiation of LSPCs? It is because hepatocyte-specific gene expression is regulated by the orchestrated action of sets of LETFs 52–65. For example, HNF4#x03B1; is known to be a key regulator of both hepatic differentiation during embryonic development and the maintenance of a differentiated phenotype in the adult liver 118. HNF4#x03B1; contributes to the regulation of a large fraction of the liver and pancreatic islet transcriptomes by binding directly to almost half of the actively transcribed genes 125. C/EBP#x03B1; maintains the differentiated state of hepatocytes and triggers the transcription of many genes expressed in the liver such as ALB and ornithine cycle enzymes involved in urea production. The conditional knockdown of C/EBP#x03B1; revealed an important role in hepatic glucose, nitrogen, bile acid and iron metabolism, all of which represent highly differentiated hepatocytic functions 126. The FOXA transcription factor homologues, formerly termed HNF3, which are expressed both in the foetal and the adult liver, seem to be involved in the expression of nearly all liver-specific genes, including tyrosine aminotransferase, phosphoenolpyruvate carboxykinase, ALB, transferrin and TTR 124–127. FOXA proteins particularly function as #x2018;pioneer#x2019; proteins to open compacted chromatin in the regulatory regions of liver-specific genes 128. Several transcription factors such as FOXA and GATA have been identified and proposed as targets of FGF and BMP signalling in early hepatic onset 129. Although these LETFs are essential for the hepatic differentiation of LSPCs, none of them is exclusively expressed in the liver; the combinatorial actions of LETFs lead to the stringency and dynamic regulation of gene expression required for the proper differentiation of LSPCs (Fig.#x00A0;4). In other words, these LETFs also interact with various other transcription factors and/or regulatory molecules in a context-dependent manner to achieve specific target gene expression.

## Conclusions and perspectives

Thus far, the clinical translation of hepatocyte transplantation as an alternative to whole liver transplantation has been hampered by the short of suitable donor organs for the isolation of transplantable hepatocytes 130. The plasticity, differentiation potential and proliferative capacity of LSPCs make them ideal candidates as alternative sources of transplantable hepatocytes 131. In the last decade, a great deal of effort and concomitant progress have been made in establishing protocols to generate mature hepatocytes *in vitro* from either pluripotent or multipotent stem cells 132,133 and, more recently, from induced pluripotent stem (iPS) cells 135,136. The differentiation of embryonic stem cells (ESCs) into mature hepatocytes has now been readily demonstrated by a number of groups 138–139. Nevertheless, the differentiation of LSPCs into hepatocytes would provide the basic rules for the generation of mature functional liver parenchymal cells. Such protocols for the hepatic differentiation of LSPCs could also provide useful *in vitro* models for studying hepatogenesis and liver regeneration 140.

To date, the molecular signals regulating the hepatic differentiation of LSPCs, which play pivotal roles in liver development, are not fully understood. The inductive protocols for the hepatic differentiation of LSPCs need to be optimized and even standardized. The most progress towards achieving this goal may come from the regulatory mechanisms of liver development and liver regeneration. To produce hepatocytes, the LSPCs should be sequentially induced to differentiate into the hepatic progenitors, the hepatic precursor cells, and finally into the functional hepatocytes, directed by the timed use of appropriate stimuli 138. The hepatic differentiation of LSPCs is a dynamically evolutionary process consisting of initiation, progression and accomplishment stages.

From the discussion of the hepatic differentiation of LSPCs, composed of current *in vitro* inductive strategies, characterization strategies, and differentiation-related mechanisms, it becomes clear that the standardization of the production of functional hepatocytes from LSPCs is the main task for the future. Here, we state several ideas that may help to guide future stem cell research: (*i*) The precise characterization of undifferentiated LSPCs and induced hepatocytes is of utmost importance for the future exploitation of stem cell technology. Phenotyping based on surface markers has thus far been insufficient. Instead, characterization should be performed at the morphological, molecular and functional levels 141. (*ii*) Liver stem/progenitor cells react differently to cytokines/GFs at successive developmental stages 16–55. The dosage, timing and combinations of cytokines/GFs should thus be fine-tuned according to the differentiated state and type of LSPC involved. Most commonly, cells exit from the cell cycle and then undergo differentiation, resulting in either a terminal, irreversible cell specialization or a particular developmental step in the life cycle #x005B;^52^, ^65^#x005D;. Hence, the dosage and combination of soluble medium additives should be fine-tuned according to this dichotomy between cell proliferation and cell differentiation. (*iii*) Finally, the molecular mechanism surrounding hepatic differentiation from LSPCs is essential for cell-based therapies. The essential factors responsible for the initiation, progression and accomplishment of hepatic differentiation should be specially considered. In detail, a more scrupulous understanding of the instructive signals emanating from the LSPC niche, together with a deeper analysis of the cell-intrinsic mechanisms governing replication- *versus* differentiation-inducing signals, is needed to reliably expand and differentiate LSPCs.

In general, an unlimited supply of stem cell-derived hepatocytes would be an invaluable resource for the development of novel cell-based therapies. Studies of the molecular mechanisms regulating the hepatic differentiation of LSPCs will facilitate the reality of such cell-based therapies for treating liver diseases. By clarifying the mechanisms of hepatic differentiation regulation, LSPC-based therapy will have a bright future.
